# ACross-Paradigm CNN–Swin Transformer Ensemble with Super-Resolution Enhancement for Multi-Class Alzheimer’s Disease Classification

**DOI:** 10.3390/bioengineering13060666

**Published:** 2026-06-08

**Authors:** Mohamed H. Habeb, Reem A. Alnanih, Lamiaa A. Elrefaei

**Affiliations:** 1 Electrical Engineering Department, Faculty of Engineering at Shoubra, Benha University, Cairo 11629, Egypt; mohamed.rehan@feng.bu.edu.eg (M.H.H.); or lamiaa.elrefaei@iu.edu.eg (L.A.E.); 2 Department of Computer Sciences, Faculty of Computing and Information Technology, King Abdulaziz University, Jeddah 21589, Saudi Arabia; 3 Software Engineering and Distributed System Research Group, King Abdulaziz University, Jeddah 21589, Saudi Arabia; 4 Faculty of Computers and Information Technology, Innovation University, 10th of Ramadan City 7055501, Egypt

**Keywords:** Alzheimer’s diagnosis, MRI classification, CNN–Swin ensemble, image super-resolution, intelligent healthcare systems engineering, vision transformers

## Abstract

Alzheimer’s disease (AD) is a global health challenge requiring early and accurate diagnosis, yet current clinical methods struggle with early stages. Deep learning approaches for MRI-based diagnosis face persistent challenges related to image quality issues, limited model generalization, and subtle inter-class variations. To address these limitations, this paper proposes a robust, end-to-end brain MRI-based framework for multi-class classification of AD stages. Positioned within the broader research priority of artificial intelligence and intelligent healthcare technologies, the proposed methodology incorporates an attention-based ensemble of deep learning models alongside an enhanced image preprocessing that uses Real-ESRGAN to mitigate common compression and resolution degradations in 2-D MRI slices. The ensemble makes use of the superior capabilities of the Swin Transformer to capture global contextual dependencies and EfficientNet-B3/MobileNetV2 for effective multi-scale feature extraction, with feature fusion performed using a Squeeze-and-Excitation attention mechanism. The experiments were performed on a publicly available Alzheimer’s MRI dataset, resulting in classification accuracy of 94.47% and 92.28% for the two proposed frameworks. The robustness and clinical interpretability of the framework are emphasized through comprehensive metrics and qualitative analysis. This framework demonstrates promising benchmark performance on a standardized public dataset, highlighting the potential of cross-paradigm ensembles combined with super-resolution preprocessing.

## 1. Introduction

Alzheimer’s disease (AD) represents one of the most severe and growing global public health concerns of the 21st century. As a progressive neurodegenerative disorder, it predominantly affects elderly populations and leads to irreversible loss of cognitive function, memory, and behavioral changes. Over 55 million individuals currently live with dementia worldwide, with Alzheimer’s contributing to nearly 60–70% of these cases as estimated by the World Health Organization (WHO). Globally, dementia ranks as the seventh leading cause of death and is a significant contributor to disability and dependency in the elderly population [1,2]. By 2050, it is expected that there will be about 153 million AD patients worldwide, which in turns indicating the critical need for early and efficient detection of AD [3]. Moreover, Alzheimer’s disease frequently remains undiagnosed in its early stages because clinical symptoms develop subtly, leading to significant neurological damage before diagnosis occurs. The traditional diagnosis of AD usually includes neuropsychological testing, clinical history, and cognitive evaluations. Additionally, clinical trials for Alzheimer’s disease (AD) continue to encounter significant challenges in patient identification, which leads to underpowered studies, longer timeframes, and rising expenses [4]. Hence, these strategies are not sensitive, scalable, or objective, particularly when the disease is in its pre-clinical or mild cognitive impairment (MCI) stages. The goal of treatment for AD is to decrease its progression because there is currently no cure. Thus, early diagnosis is essential for slowing symptom progression, enhancing patient care options, and enabling different timely intervention strategies. This also enhances patients’ quality of life and facilitating better care when cognitive capacities deteriorate.

The availability and widespread use of medical imaging techniques, Magnetic Resonance Imaging (MRI), PET, and single-photon emission computed tomography (SPECT), have enabled significant developments in Alzheimer’s diagnosis and prognosis [5]. Research on Alzheimer’s disease prediction has also benefited from the availability of datasets for patients with Alzheimer’s disease (AD), cognitive normal (CN), and mild cognitive impairment (MCI) that include neuroimaging, genetic, and demographic modalities. These datasets can be analyzed using deep learning techniques, which is the most widely used technique for developing diagnostic models from medical imaging data.

Nowadays, Magnetic Resonance Imaging (MRI) remains the one of the most widely used non-invasive techniques for detecting structural brain abnormalities related to Alzheimer’s. MRI provides detailed visualization of cortical thinning, hippocampal atrophy, and ventricular enlargement—hallmark indicators of AD [6]. Nevertheless, MRI suffers from practical and clinical challenges, including motion artifacts, image noise, low contrast, inter-scanner variability, and diagnostic inconsistencies due to the dependence on visual interpretation of observers [7]. Moreover, the availability of expert radiologists is limited, especially in resource-constrained environments. This makes it necessary to explore automated diagnostic solutions that are both efficient and accurate. In this context, machine learning, especially deep learning (DL) methodologies, has emerged as a compelling solution to automate MRI-based diagnosis, reducing human dependency while improving detection sensitivity and reproducibility. Their ability to learn hierarchical representations directly from raw image pixel intensities eliminates the need for manual feature engineering, making them ideal for complex classification tasks like AD detection. To reliably classify MRI scans into Alzheimer’s stages, several convolutional neural networks (CNNs) architectures have been used in the field of Alzheimer’s research, including VGG, ResNet, and DenseNet. However, the requirement for large, labeled datasets and the high computing cost have presented significant challenges to their clinical integration. Another problem is the gap between binary and multi-class performances, which indicates that current models still face difficulties in differentiating the subtle morphological changes between AD stages [8].

A major limitation that poses challenges to robust deep learning models training for AD detection is the poor image quality, which appears in low contrast, noise, and resolution loss. These imaging issues can hide or distract from subtle pathological changes in brain tissue, leading to decreased model sensitivity. To address the limitations of low-resolution and noisy MRI scans commonly encountered in clinical settings, this study incorporates Real-ESRGAN (Real-World Enhanced Super-Resolution GAN), which is an advanced deep learning model capable of reconstructing high-quality images from real-world degraded inputs. Unlike traditional super-resolution models trained on synthetically down-sampled data, Real-ESRGAN is designed to handle unknown and complex degradation patterns, making it well-suited for enhancing medical images used in Alzheimer’s diagnosis.

While individual deep learning models such as CNNs and transformers have shown great promise in Alzheimer’s diagnosis, but ensemble learning strategies have emerged as powerful tools to improve diagnostic robustness and generalization. In this study, we propose an ensemble framework that integrates the strengths of two advanced deep learning architectures: MobileNetV2, known for its efficiency and fine-grained texture extraction, and EfficientNet-B3, also utilized for its state-of-the-art efficiency and superior multi-scale feature representation capabilities, with the Swin Transformer, which captures long-range dependencies and contextual relationships. These two branches, either MobileNetV2 or EfficientNet-B3 with the Swin Transformer, are fused using a Squeeze-and-Excitation (SE) attention mechanism, which is applied independently to each branch’s extracted feature vectors immediately after extraction but prior to concatenation. This channel-wise recalibration occurs at the feature level, allowing the ensemble to emphasize the most discriminative features from each model. This attention-guided fusion not only mitigates the risk of redundant or conflicting feature contributions but also enhances the overall classification accuracy and model interpretability in complex multi-stage Alzheimer’s detection tasks. In addition, we employ a strong evaluation approach based on k-fold cross-validation during training, in addition to a soft-voting ensemble inference across folds, to ensure model generalization and stability across patient variability. This enables the final choice to represent agreement across many training perspectives. This setup mitigates overfitting, enhances robustness, and improves classification performance in the presence of subtle inter-class differences in Alzheimer’s stages.

In summary, this study introduces a robust, end-to-end deep learning framework for automated multi-class classification of Alzheimer’s disease using structural brain MRI. The approach focuses on enhancing real-world clinical applicability by integrating advanced image preprocessing, model ensembling, and rigorous evaluation protocols. The classification component leverages an ensemble of MobileNetV2 or EfficientNet-B3 with Swin Transformer architectures, fused through a Squeeze-and-Excitation (SE) attention mechanism, which adaptively emphasizes the most discriminative features from each model branch. To promote generalization and reduce variance, the framework is trained using 5-fold cross-validation, and final predictions are made via soft voting across the five trained folds, capturing the consensus from multiple perspectives. Experiments were conducted on a publicly available MRI datasets: Alzheimer’s MRI 4-Class Dataset from Kaggle, comprising T1-weighted scans labeled across Alzheimer’s disease stages. The model was evaluated using comprehensive metrics, including accuracy, precision, recall, F1-score, and confusion matrices, to validate its performance. The main contributions of this research are:

• Integration of Real-ESRGAN for MRI enhancement under real-world degradation conditions;
• Design of a lightweight attention-based ensemble combining MobileNetV2/EfficientNet-B3 and Swin Transformer for improved multi-class classification;
• Implementation of 5-fold cross-validation with soft-voting inference, promoting stable and reliable predictions;
• Evaluation across a publicly available Alzheimer’s MRI dataset, demonstrating generalizability;
• A comparative analysis of enhanced vs. raw MRI inputs, highlighting the diagnostic benefits of super-resolution preprocessing.

By addressing challenges such as image degradation, model generalization, and clinical interpretability, this work contributes a robust and accurate framework for Alzheimer’s diagnosis that holds promise for real-world and telemedicine applications.

The remainder of this paper is organized as follows: Section 2 reviews related deep learning approaches for Alzheimer’s disease detection. Section 3 describes the proposed methodology, including data preprocessing, model architecture, and the ensemble fusion strategy. Section 4 outlines the experimental setup and evaluation metrics. Section 5 presents and discusses the results, including quantitative and qualitative performance comparisons, and limitations. Finally, Section 6 concludes the paper with the key findings, overall contributions of the study, and future research directions.

## 2. Related Work

This section reviews recent advances in deep learning-based Alzheimer’s disease (AD) classification, highlighting architectural trends, ensemble strategies, image enhancement techniques, and evaluation methodologies. The discussion aims to identify existing strengths and unresolved challenges that motivate the proposed framework.

### 2.1. Deep Learning for Alzheimer’s Classification

The application of deep learning (DL) to Alzheimer’s disease (AD) has evolved through last decade to address many core challenges such as accuracy, efficiency, and clinical applicability. Early work established the efficacy of standard convolutional neural networks (CNNs) like VGGNet and ResNet for binary classification passing by the ensemble and fusion techniques towards the generative and most recent attention mechanisms.

Concurrently, ensemble learning has emerged as a dominant strategy to enhance model robustness and generalization. Demonstrating the effectiveness of model fusion, the proposed work in [6] achieved AD classification performance about 99.8% accuracy by integrating DenseNet121 and Xception net into a hybrid CNN-based model. The approach, which also included SMOTE for class balancing, emphasized the ensemble robustness from pretrained networks for medical image analysis. The authors in [9] addressed the High Dimension Low Sample Size (HDLSS) problem in AD staging by developing a Deep Belief Network (DBN) with Restricted Boltzmann Machines for multi-modal data fusion, besides using a two-tiered feature selection process and PCA for dimensionality reduction. Their framework integrated MRI features (OASIS-4), genetic biomarkers, and cognitive scores and the model achieved accuracy 98.79% for MCI vs. AD, which demonstrated the effectiveness of multi-task learning on heterogeneous data. In addition, the work introduced in [10], proposed a deep ensemble framework for AD classification from multisource clinical data (NACC UDS). In this work sparse autoencoders were utilized for feature fusion, with a diverse set of base classifiers, and a Deep Belief Network, as a meta-classifier that was optimized with cost-sensitive learning. The framework outperformed traditional ensemble methods and showed a high recall rate, which is critical for screening in primary care settings. Another work [11], which proposed an MRI-based ensemble model of VGG16 and EfficientNet-B2 for multi-class AD classification. The authors utilized the ADASYN oversampling technique to address the issue of dataset imbalance. The proposed model achieved an accuracy of 97.35%, and AUC of 99.64% on Kaggle datasets. This demonstrates that fusing two pretrained CNNs can yield robust results superior to individual models. On the other hand, a longitudinal prediction framework for AD progression was developed by [12]. It models atrophy trajectories in hippocampal and ventricular ROIs from T1-MRI (ADNI/AIBL) using Linear Mixed Effects models. The features derived from these trajectories were classified via an ensemble of SVM and Logistic Regression, achieving accuracies of 71% for predicting CN to MCI and 78% for predicting MCI to AD. This approach provides a flexible, feature-engineered method for early risk assessment from clinical-timepoint data.

More powerful deep architectures were developed such as in [7], where the authors introduced a hybrid 3D-CNN and RNN (Bidirectional-GRU) architecture with attention for multi-modal AD classification, where structural MRI and fMRI data was integrating with clinical variables. Using transfer learning from large-scale datasets and processing ADNI data, their model achieved a mean accuracy of 99.5% in distinguishing NCI, MCI, SCI, and AD classes. While in [13], a 3D densely connected CNN was developed with a novel connection-wise attention mechanism (CAM-CNN) for AD and MCI conversion prediction from T1-MRI. The model introduced a high accuracy of 97.35% for AD vs. NC using 3D patches from ADNI scans that demonstrated the efficacy of attention mechanisms for weighing multi-level spatial features in volumetric data. Furthermore, the framework in [14] addressed a critical challenge in applying deep learning to real-world clinical MRI by developing an advanced data matching algorithm to control for technical and demographic confounders. Using a dataset from the Mass General Brigham system, an ensemble of 3D ResNet-50 models achieved an AUROC of 0.82 for classifying AD/MCI vs. healthy controls, highlighting the importance of confounder mitigation for generalizable models.

For exploring non-Euclidean data representations, the authors in [1] applied a Graph Convolutional Network (GCN) with a self-attention pooling mechanism to classify AD using structural brain networks derived from Diffusion Tensor Imaging (DTI). By representing the brain regions as nodes (AAL atlas) and Fractional Anisotropy (FA) as edge weights, the model achieved 87.5% accuracy on ADNI data. This work demonstrates the potential of GCNs for directly learning from connectome data while identifying task-relevant brain regions. While in [15], the authors proposed FSNet, which is a dual-interpretable Graph Convolutional Network that simultaneously selects and weights important features and samples for AD classification from T1-MRI. As in [1], graphs were constructed from brain region features and then Hadamard product was applied with learned weight matrices. The FSNet introduced outstanding state-of-the-art performance on ADNI binary classification tasks while providing interpretable rankings of relevant brain regions.

The work in [16] demonstrated the efficacy of transfer learning (TL) for multi-class AD classification from MRI, employing a fine-tuned EfficientNet-B0 model on ADNI axial-plane images. This approach yielded accuracy of 98% on test data with minimal computational cost. A comprehensive pipeline for multi-class AD staging from MRI was proposed by [17], featuring a Multi-Scale Pooling Residual Autoencoder for white matter segmentation, a preprocessing for denoising and enhancement, and subsequent classification using an RBF-SVM. Their system achieved 99.14% segmentation and classification accuracy with 99.53% for AD vs. NC on ADNI, which illustrated the value of specialized preprocessing and segmentation for feature extraction. In addition, an optimized VGG-16 architecture for AD staging was introduced [18], where hyperparameters were tuned using an Arithmetic Optimization Algorithm (AOA). By applying it to T1-MRI form ADNI and OASIS, the model achieved high accuracy of 97% for normal class with reduced computational cost. Another work based on deep learning (DL) and machine learning (ML) method for diagnosing AD was introduced by [19]. The method adopted EfficientNet-B0 and a constructed CNN complemented by ML classifiers and worked on clinical test data and MRI images. For interpretability, Gradient-weighted Class Activation Mapping (Grad-CAM) and Local Interpretable Model-agnostic Explanations were utilized.

The field subsequently progressed towards developing more efficient architectures, such as lightweight CNNs for resource-constrained environments. In [20], the authors developed an automated framework for AD classification from MRI using a Particle Swarm Optimization (PSO) algorithm to design a lightweight CNN. Their model, trained and validated on Kaggle and ADNI datasets, achieved an accuracy of 99% and F1-scores, which focused on the potential of architecture search for efficient diagnostic models. Another work by [21], which developed a hybrid CNN-PSO framework for medical image classification for AD detection and brain tumor diagnosis. In this work, PSO automated the selection of optimal CNN hyperparameters (filter number/size, pooling stride), achieving 98.5%accuracy on AD datasets and outperforming standard transfer learning models. Another impressive work was introduced by [22], in which lightweight CNN and YAMNet were adopted in dual-stream multi-modal framework that transformed MRI images into audio signals by a multi-scale, multi-orientation Gabor filtering. Then the audio representations are fused via logistic regression with those 2-D MRI slices, achieving accuracy of 98.2% in classifying AD from CN cases, 94% for AD vs. MCI, and 93.2% for MCI vs. CN.

### 2.2. Related Work Summary and Research Gap

Despite significant advances in AD detection/classification frameworks and models, critical limitations still exist in the model architecture, data robustness, and evaluation methodology. First, although ensembles improve performance, the majority of frameworks fuse similar CNN variants, neglecting the complementary potential of different paradigms like efficient CNNs and global-context transformers. Second, more focus is needed on the impact of advanced deep learning-based image enhancement on modern ensemble performance. Third, stability improvements from cross-validation fold-level ensemble inference are overlooked when relying on single best-model evaluation.

To address these gaps, we hypothesize that a cross-paradigm ensemble, fed with enhanced images and assessed using a strong fold-wise consensus, will achieve superior and more generalizable multi-class AD classification. We test this by proposing a novel framework that integrates:

1. Real-ESRGAN-based super-resolution enhancement to mitigate 2-D MRI slices degradation;
2. A dual-stream ensemble combining the computational efficiency of MobileNetV2 and EfficientNet-B3 with the global contextual modeling capability of a Swin Transformer, fused through a (SE) attention mechanism;
3. A soft-voting inference strategy across cross-validation folds to enhance decision stability and reliability.

The proposed framework is rigorously evaluated on the Alzheimer’s MRI 4-Class Dataset to quantify the impact of image enhancement, architectural complementarity, and fold-level consensus on robust multi-class AD classification performance.

## 3. Methodology

**Figure 1:**
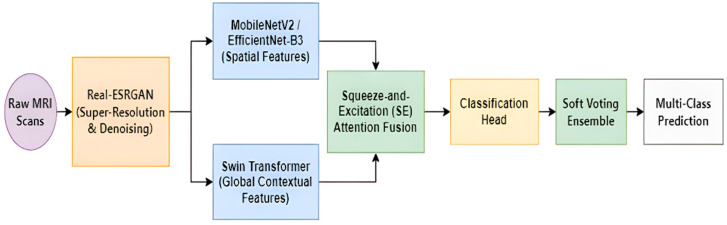
Schematic representation of the proposed deep learning framework for multi-class Alzheimer’s disease stage classification.

This section outlines the proposed deep learning framework designed for robust and accurate multi-class classification of Alzheimer’s disease stages using structural MRI scans. The pipeline, as shown in Figure 1, integrates a hybrid ensemble architecture that combines CNN and transformer-based vision models—MobileNetV2/EfficientNet-B3 and Swin Transformer—with a prior Real-ESRGAN-based super-resolution enhancement stage. To improve feature fusion and channel-wise relevance, a Squeeze-and-Excitation (SE) attention mechanism is employed, allowing the model to dynamically adjust feature importance. The architecture is trained and evaluated using the Alzheimer’s MRI 4 Classes dataset. We describe the complete methodology in the following subsections, including image enhancement, model design, attention-based fusion, training configuration, and evaluation protocols, aiming to improve diagnostic accuracy across diverse stages of cognitive impairment.

### 3.1. Dataset

This study utilizes the Kaggle brain MRI dataset [23] for training and evaluating the proposed multi-class Alzheimer’s classification framework. The dataset comprises a total of 6400 T1-weighted brain MRI slices, categorized into four classes corresponding to progressive stages of Alzheimer’s disease. The class distribution is as follows: Non-Demented (3200 images), Very Mild Demented (2240 images), Mild Demented (896 images), and Moderate Demented (64 images). All images are 2-D axial slices provided in JPEG format and have been preprocessed to a uniform resolution of 176 × 208 pixels. As illustrated in Table 1, the dataset is notably imbalanced, with a severe underrepresentation of the Moderate Demented class, which constitutes only 1% of the total data. This distribution reflects a realistic clinical challenge, as advanced dementia cases are often less frequently captured in such datasets. However, this imbalance poses a serious problem for deep learning models, which may become biased toward the majority classes (Very Mild Demented and Non-Demented) and find it difficult to differentiate between the subtle and significant features of the mid-to-late disease stages. Due to the absence of subject-level metadata in the public Kaggle dataset, partitioning was performed at the slice level using stratified 5-fold cross-validation. To prevent information leakage, all data augmentations were applied strictly within training folds after partitioning, and no augmented samples were permitted in validation or test sets.

### 3.2. Real-ESRGAN

**Figure 2:**
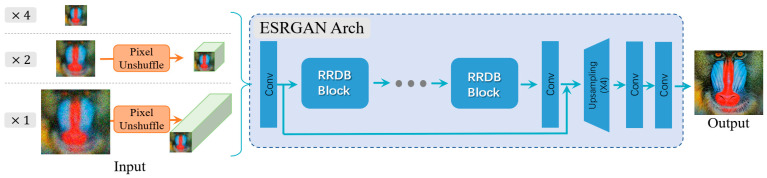
Architecture of the Real-ESRGAN model [24].

This study incorporates Real-ESRGAN (Real-World Enhanced Super-Resolution Generative Adversarial Network) [24] for low-quality MRI scans enhancement before the features extraction process. Real-ESRGAN builds upon the Enhanced SRGAN (ESRGAN) architecture, advancing its robustness for blind super-resolution in real-world scenarios. ESRGAN was mainly trained on bicubic degradation, but Real-ESRGAN introduces a generalized synthetic degradation pipeline during training. This enabled it to model realistic distortions such as blur, noise, compression artifacts, and non-uniform degradation patterns common in clinical 2-D MRI lossy JPEG slices. For stabilizing training and preserving deep features, the generator architecture retains the Residual-in-Residual Dense Block (RRDB) backbone, as shown in Figure 2. However, Real-ESRGAN improves the discriminator by using a U-Net-based structure, which allows for more localized discrimination and greater attention to texture-level realism. The training objective in Real-ESRGA integrates pixel-wise loss, perceptual loss from a pretrained VGG network, and enhanced adversarial loss to optimize fine detail reconstruction and minimize artifacts. A High-Order Degradation Model effectively simulates intricate degradations during training with synthetic data, ensuring adaptability to real-world images. In this study, we utilized the pretrained Real-ESRGAN generator for inference-only enhancement of MRI slices, rather than retraining the model on our dataset. This choice balances methodological rigor with practical constraints, leveraging the model’s demonstrated ability to handle complex, real-world degradations while avoiding overfitting to the limited Alzheimer’s MRI samples. The Real-ESRGAN pipeline is shown in Algorithm 1 (Note: In this study, we applied the pretrained Real-ESRGAN generator in inference-only mode without additional training or fine-tuning on the Alzheimer’s dataset). The application of Real-ESRGAN to medical imaging has been recently validated by [25], who demonstrated its effectiveness across four medical modalities including 2-D sliced brain MRI (BraTS dataset), achieving PSNR of 36.99 dB and SSIM of 0.97. Their findings support the feasibility of Real-ESRGAN from natural to medical images. In the context of this study, Real-ESRGAN is applied as a preprocessing step to enhance the MRI slices. By suppressing noise and enhancing spatial fidelity, Real-ESRGAN improves the signal quality presented to downstream deep learning tasks, potentially increasing diagnostic accuracy in nuanced pathological cases. The integration of Real-ESRGAN into the pipeline explicitly tackles picture deterioration, a frequently neglected aspect in previous AD classification research, therefore enhancing model robustness under realistic imaging settings.
**Algorithm 1:** Overview of Real-ESRGAN Training Procedure**Input:**•$I_{L}$: Synthetically degraded low-resolution image•$I_{H}$: Corresponding high-resolution image•G: Generator network (RRDB backbone)•D: U-Net discriminator•$\lambda_{adv},\;\lambda_{pix},\;\lambda_{perc}$: Loss weights**Output:** $G_{trained}$: Trained generator capable of producing super-resolved image Initialize generator parameters θInitialize discriminator parameters ϕ**Stage I: Generator Warm-Up Pretraining**3.For epoch = 1 to $n_{1}$ do4.  For each batch $\left(I_{L},I_{H}\right)$ do5.    Generate super-resolved image:6.       $I_{SR}\leftarrow G\left(I_{L}\right)$7.    Compute pixel-wise L1 loss:8.      $L_{pix}\leftarrow ||I_{H}-I_{SR}|{|}_{1}$9.    Update generator parameters to minimize $L_{pix}$
10.  End for11.End for**Stage II: Adversarial Fine-Tuning**12.For epoch = 1 to $n_{2}$ do13.  For each batch $\left(I_{L},I_{H}\right)$ do**A.** **Discriminator Update (Freeze Generator)**14.    Generate super-resolved image (detach gradients):15.      $I_{SR}\leftarrow G\left(I_{L}\right)$16.    Compute relativistic discriminator loss $L_{D}$ using RaGAN formulation17.    Update discriminator parameters to minimize $L_{D}$
**B.** **Generator Update (Freeze Discriminator)**18.    Generate super-resolved image:19.      $I_{SR}\leftarrow G\left(I_{L}\right)$20.    Compute adversarial loss $L_{adv}$21.    Compute pixel + frequency reconstruction loss $L_{pix}$22.    Compute perceptual loss $L_{perc}$23.    Compute total generator loss:24.      $L_{G}\leftarrow \lambda_{adv}L_{adv}+\lambda_{pix}L_{pix}+\lambda_{perc}L_{perc}$25.    Update generator parameters to minimize $L_{G}$26.  End for27.End for28.Return trained generator $G_{trained}$

### 3.3. Deep Feature Extraction

In modern systems for neurodegenerative diseases like Alzheimer’s disease (AD), the success of a classification framework depends largely on the quality of features taken from brain MRI scans. Deep convolutional neural networks (CNNs) and transformer-based models have become key methods for automatic representation learning. In this work, we used the strengths of MobileNetV2, a lightweight CNN, EfficientNet-B3, and the Swin Transformer, a hierarchical vision transformer, to extract deep spatial and contextual features from improved MRI slices. We then combined these features using a Squeeze-and-Excitation (SE) attention mechanism to create a detailed and effective representation for final classification.

#### 3.3.1. MobileNetV2 for Lightweight Spatial Feature Extraction

Deep learning-based neuroimaging pipelines must balance model accuracy, interpretability, and computational efficiency. This is particularly important in situations with limited computational resources, like real-time clinical inference or edge deployment. To address this, we used MobileNetV2 [26] as a lightweight and effective Convolutional Neural Network (CNN) backbone to extract spatially localized features from enhanced 2-D MRI slices. MobileNetV2 is built on a key innovation: the inverted residual block with linear bottlenecks. This design helps the network maintain its ability to represent information while significantly lowering the number of parameters and computational demands. Unlike traditional residual blocks, which expand after applying non-linearity, MobileNetV2 first expands the input features using a 1 × 1 convolution. It then uses depth-wise separable convolution to learn spatial features and projects the output back to a lower-dimensional space with another 1 × 1 convolution, without applying non-linearity at the bottleneck output. This setup preserves information and reduces overfitting, which is particularly helpful in medical imaging where datasets are often small, and over-parameterization can harm performance. The network primarily uses depth-wise separable convolutions. This breakdown a standard convolution into two parts: a depth-wise convolution (spatial filtering for each channel) and a pointwise convolution (1 × 1 convolution across channels). This architecture greatly decreases both FLOPs and memory use while maintaining high accuracy. Therefore, MobileNetV2 is well-suited for fast inference and deployment on resource-limited clinical devices, such as portable imaging analysis units or telehealth platforms. In our proposed ensemble framework, the MobileNetV2 backbone processes each pre-enhanced MRI slice and produces a high-dimensional feature representation $F_{M}\in {\mathbb{R}}^{1280}$ from the final global average pooling layer. These features capture essential localized anatomical details that are crucial for differentiating between subtle stages of Alzheimer’s (non-dementia, very mild, mild, and moderate dementia), where minor variations in brain structure can significantly affect diagnosis. Additionally, MobileNetV2’s small model size and rapid processing speed make it ideal for training and inference on multiple folds during cross-validation. This step is vital for our system, which uses ensemble averaging to ensure strong performance.

The integration of MobileNetV2 with the Swin Transformer, described later in Section 3.3.3, is intentionally designed to capture complementary pathological signatures. MobileNetV2 efficiently extracts localized features, while the Swin Transformer models distributed neurodegenerative patterns that CNN receptive fields often miss. By fusing these streams via independent SE attention, our framework explicitly addresses the spatial-scale mismatch in single-architecture AD classifiers. This design enhances robustness in differentiating early-to-late disease stages without inflating computational cost to be suitable for resource-constrained clinical.

#### 3.3.2. EfficientNet-B3 for Scalable and Accurate Feature Extraction

In addition to lightweight backbones such as MobileNetV2, our framework incorporates EfficientNet-B3 [27] a deep convolutional architecture known for its compound scaling strategy. This strategy balances network depth, width, and input resolution in a careful way. Unlike traditional methods that scale only one dimension, such as depth or width, EfficientNet uses a compound coefficient to uniformly scale all three. As a result, it achieves top performance with significantly fewer parameters and FLOPs compared to typical CNNs. This balance of efficiency and accuracy makes EfficientNet-B3 especially suitable for neuroimaging applications. Subtle pathological changes require detailed feature extraction from high-resolution MRI slices without incurring high computational costs. The higher input resolution typically used with EfficientNet-B3, helps the model retain intricate variations that lower-resolution architectures may miss. In our ensemble pipeline, EfficientNet-B3 works as a strong feature extractor that produces a compact yet highly discriminative representation $F_{E}\in {\mathbb{R}}^{1536}$. EfficientNet-B3 shows resilience against overfitting by using its depth-wise convolutions and recalibration, which help reduce sensitivity to noise and inter-scanner variability, common issues in multi-center MRI datasets. This representation increases sensitivity to both more general morphological patterns linked to mild AD stages and localized structural deterioration. The utilization of EfficientNet-B3 allows evaluation of a performance-oriented ensemble variant alongside the lightweight configuration.

Pairing EfficientNet-B3 with the Swin Transformer leverages compound scaling to preserve multi-resolution morphological details alongside global contextual modeling. EfficientNet-B3’s hierarchical feature maps capture both gross anatomical changes and fine-grained feature texture variations, while the Swin branch resolves long-range anatomical dependencies. Then, the SE-based fusion dynamically recalibrates channel importance, to ensure that the ensemble prioritizes the most discriminative cross-paradigm representations for each AD stage.

#### 3.3.3. Swin Transformer for Hierarchical Contextual Modeling in Brain MRI

Convolutional neural networks (CNNs), like MobileNetV2, are good at finding basic spatial and texture patterns in brain MRI images. However, their limited scope and focus on local areas can make it hard to understand overall relationships, which are important for medical imaging tasks like classifying Alzheimer’s disease (AD). To deal with this, we use the Swin Transformer [28], a vision transformer designed to efficiently model global context in high-resolution image data, and can be scaled up easily.

**Figure 3:**
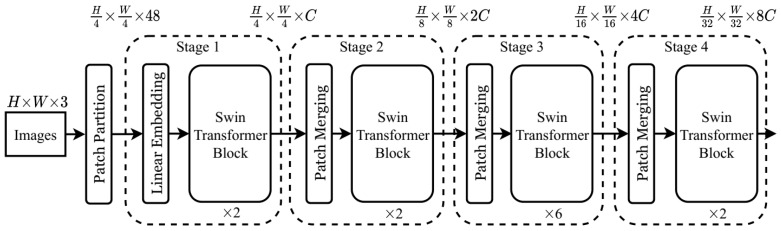
Swin Transformer pipeline overview [28].

The Swin Transformer starts by dividing the image into fixed-size patches, treating each one as a token, like the vision transformer (ViT). But the Swin Transformer goes beyond ViT’s design by using hierarchical features and a shifted window self-attention method. This structure not only mimics the multi-scale behavior of CNNs but also greatly increases how well it computes and models global features. Each stage of the Swin Transformer has Window-based Multi-head Self-Attention (W-MSA) and Shifted Window Multi-head Self-Attention (SW-MSA) modules. The changing window shift pattern lets the model make connections between windows that would be missed in normal window-based attention. This allows for long-range spatial interactions while keeping computational complexity linear relative to image size. The transformation process within each block is defined as:

(1) $${\hat{z}}^{l}=W-MSA\left(LN\left(z^{l-1\;}\right)\right)+\;z^{l-1},$$

(2) $$z^{l}=MLP\left(LN\left({\hat{z}}^{l}\right)\right)+{\hat{z}}^{l},$$

(3) $${\hat{z}}^{l+1}=SW-MSA\left(LN\left(z^{l\;}\right)\right)+z^{l\;},$$

(4) $$z^{l+1}=MLP\left(LN\left({\hat{z}}^{l+1}\right)\right)+{\hat{z}}^{l+1},$$

where ${\hat{z}}^{l}$ and $z^{l}$ denote the output features of the (S)WMSA module and the MLP module for block l, respectively;

Through hierarchical patch merging, the Swin Transformer progressively constructs multi-scale feature maps (e.g., 1/4, 1/8, 1/16, and 1/32 of the input resolution). This pyramid structure enables effective modeling of distributed structural variations such as hippocampal atrophy, cortical thinning, and ventricular enlargement that are key biomarkers of Alzheimer’s progression. The final contextual embedding extracted from the Swin encoder is $F_{S}\in {\mathbb{R}}^{1024}$. This representation captures long-range structural dependencies and hierarchical anatomical relationships within each MRI slice. As illustrated in Figure 3 [28], the Swin Transformer pipeline consists of patch embedding, successive hierarchical Transformer stages with W-MSA and SW-MSA operations, progressive patch merging, and final high-level contextual feature extraction.

Following the Swin Transformer encoding stage, we proposed two ensemble configurations to evaluate the trade-off between computational efficiency and representational capacity:

1. Framework A (Lightweight Ensemble): Integrates the MobileNetV2 backbone with the Swin Transformer encoder;
2. Framework B (High-Capacity Ensemble): Integrates the EfficientNet-B3 backbone with the Swin Transformer encoder.

In both configurations, each enhanced MRI slice is processed in parallel by a CNN backbone and the Swin Transformer. The CNN branch produces a localized spatial descriptor either MobileNetV2 or EfficientNet-B3, while the Swin Transformer generates contextual embedding. These heterogeneous feature representations are subsequently integrated using an attention-based fusion mechanism (described in Section 3.3.4).

#### 3.3.4. Attention-Based Feature Fusion

To integrate heterogeneous feature representations, a Squeeze-and-Excitation (SE) attention mechanism is applied independently to each feature branch before concatenation. Given the feature vectors extracted from the CNN backbone ($F_{CNN}$) and the Swin Transformer ($F_{S}$), where $F_{CNN}=F_{M}$ for MobileNetV2 or $F_{CNN}=F_{E}$ for EfficientNet-B3, and $F_{S}$ denotes the Swin Transformer embedding.

Each SE block performs: Global average pooling (squeeze), Bottleneck fully connected layers with ReLU and sigmoid activations, and channel-wise recalibration (excitation). This produces recalibrated feature vectors: ${\tilde{F}}_{CNN},{\tilde{F}}_{S}$. The fused embedding is then formed by concatenation:

(5) $$F_{fusion}=\left[{\tilde{F}}_{CNN}∥{\tilde{F}}_{S}\right],$$

Finally, the fused representation is passed to a fully connected classification head for four-class Alzheimer’s disease stage prediction.

### 3.4. Framework Advantages for AD Classification

The proposed dual-encoder ensemble architecture is designed to balance computational efficiency and representational capacity in multi-class Alzheimer’s disease staging. Hence, two configurations are evaluated to accommodate different deployment constraints: a lightweight configuration integrating MobileNetV2 with the Swin Transformer, and a higher-capacity configuration integrating EfficientNet-B3 with the Swin Transformer. The convolutional backbone (MobileNetV2 or EfficientNet-B3) extracts localized spatial representations, while the Swin Transformer models hierarchical and long-range contextual dependencies. This complementary design enables multi-scale feature integration across local and global anatomical structures. Furthermore, the incorporation of Squeeze-and-Excitation (SE) based feature recalibration ensures adaptive channel-wise weighting before fusion. This mechanism facilitates dynamic integration of heterogeneous feature streams without increasing architectural complexity. By combining efficient convolutional encoding with hierarchical self-attention modeling, the proposed framework provides a structurally diverse representation of MRI data suitable for robust multi-stage Alzheimer’s classification.

## 4. Experimental Setup

The following details the comprehensive experimental protocol designed to rigorously evaluate the proposed hybrid ensemble framework for multi-stage Alzheimer’s disease classification. It demonstrates the dataset characteristics, preprocessing and super-resolution enhancement strategy, model architecture configurations, training setup, cross-validation methodology, ensemble voting mechanism, and evaluation metrics. In addition, considerable attention is given to addressing class imbalance while ensuring robust generalization through stratified cross-validation and adopting comprehensive performance measures suitable for imbalanced medical imaging tasks. The experimental design aims to provide a clear, reproducible, and clinically relevant assessment of the proposed CNN–Transformer ensemble models.

### 4.1. Dataset Preparation

To develop and validate the proposed Alzheimer’s diagnostic framework, we utilized a publicly available benchmark dataset comprising structural MRI scans showing different stages of cognitive deterioration. Each image was preprocessed, enhanced, and fed into a unified ensemble classification model, allowing strong data evaluation. The Alzheimer MRI 4 Classes dataset contains 6400 T1-weighted axial MRI images, sorted into four medical groups: Non-Demented (3200 images), Very Mild Dementia (2240 images), Mild Dementia (896 images), and Moderate Dementia (64 images). This dataset naturally has uneven distribution, because of the limited number of moderate cases that reflect class distributions in real life. All images were standardized to a spatial resolution of 224 × 224 pixels, to ensure compatibility with the pretrained backbones, and then normalized. To increase generalization, data augmentation methods like rotation, intensity shifting, and random horizontal flipping were used during training.

Due to both the lack of subject-level metadata in the public Kaggle dataset and the Moderate Dementia class, which comprises only 64 slices derived from two subjects, strict patient-level splitting is statistically unfeasible without disrupting stratified cross-validation. Consequently, partitioning was performed at the slice level using stratified 5-fold cross-validation. To prevent information leakage, all data augmentations were applied strictly within training folds after partitioning, and no augmented samples were permitted in validation or test sets. While slice-level CV may slightly overestimate generalization compared to patient-level protocols, we mitigate this risk through conservative evaluation practices, including fold-wise soft-voting inference and comprehensive imbalance-aware metrics (MCC and Kappa).

### 4.2. MRI Image Enhancement

Structural MRI scans often suffer from noise, blurriness, and resolution degradation due to motion artifacts, hardware limitations, and varying acquisition protocols. To overcome these difficulties and improve the precision of brain features relevant to Alzheimer’s staging, we adopted the Real-ESRGAN-based super-resolution framework in which each MRI slice was processed through a pretrained Real-ESRGAN generator to produce an enhanced high-resolution image with reduced noise and improved structural clarity. The resulting enhanced images were then used as inputs to the deep learning classification pipeline.

### 4.3. Model Implementation

The proposed diagnostic two framework uses a hybrid dual-branch ensemble architecture, composed of MobileNetV2 and the Swin Transformer while the other constituted of EfficientNetB3 and the Swin Transformer, fused via a Squeeze-and-Excitation (SE)-based attention mechanism, to efficiently capture both local and global anatomical features from brain MRI scans. This combination balances between efficiency, accuracy, and representational diversity, which is a crucial aspect for identifying heterogeneous patterns of Alzheimer’s disease progression. The utilized backbone feature extractors are as follows:

• MobileNetV2: A depth-wise separable convolutional network. MobileNetV2 was used to extract fine-grained local features from MRI slices. The classifier head was removed, and the feature layers were used directly. The output of this branch is a 1280-dimensional vector per input image;
• EfficientNet-B3: Optimized via compound scaling for balanced depth, width, and resolution. EfficientNet-B3 was employed to extract hierarchical multi-scale features from MRI slices. The original classification head was removed, and the final convolutional block was used as the feature extractor. The output of this branch is a 1536-dimensional feature vector per input image;
• Swin Transformer: Characterized by its hierarchical self-attention and shifted windows, selected for its strong ability to model long-range dependencies and hierarchical spatial representations. The forward_features output is extracted from the final transformer block, yielding a 1024-dimensional feature vector.

The pretrained models were fine-tuned during training using the utilized Alzheimer’s dataset. The pretrained weights helped accelerate convergence and improve generalization, especially given the relatively limited dataset sizes in the medical domain. To combine the heterogeneous output features from both branches, we introduced an SE-based attention fusion module. Each feature vector (from MobileNet/EfficientNet and Swin) is passed through an independent Squeeze-and-Excitation block, consisting of:

• Global average pooling;
• Two fully connected layers with ReLU and sigmoid activations;
• Channel-wise feature recalibration.

Then, the recalibrated feature vectors were concatenated and forwarded to a fully connected classification head composed of:

• Linear layer with ReLU activation;
• Dropout layer;
• Final linear layer with Softmax activation.

The output layer produces a four-class probability distribution corresponding to the Alzheimer’s disease stages. Based on that, a training configuration for the framework was carefully investigated, and hence, the model was trained under the following settings:

• Loss Function: Cross-entropy loss;
• Optimizer: Adam optimizer with initial learning rate $1\times 10^{-4}$, reduced on plateau;
• Batch Size: 16;
• Epochs: 50;
• Regularization: Dropout;
• Early Stopping: Based on validation loss (patience = 5 epochs);
• Mixed Precision Training: Enabled using PyTorch autocast and GradScaler.

All experiments were conducted on an NVIDIA GeForce RTX 3080 GPU (16 GB VRAM) using PyTorch 2.4.0 and Python v3. The average training time per fold was approximately 58–60 min.

### 4.4. Cross-Validation and Voting Ensemble Strategy

Ensuring the integrity of predictive models and reducing the probability of overfitting is critically significant in the field of medical image classification, especially when addressing relatively limited datasets and subtle class differentiations, as illustrated in the categorization of stages of Alzheimer’s disease. To alleviate these issues, we implemented a stratified k-fold cross-validation methodology alongside a fold-wise soft-voting ensemble approach during the inference phase.

#### 4.4.1. K-Fold Cross-Validation for Training

A 5-fold stratified cross-validation protocol was employed to ensure robust performance estimation while preserving class distribution across folds. The dataset was partitioned into five subsets, each maintaining proportional representation of the four Alzheimer’s stages. This stratification is particularly important given the severe class imbalance. For each of the two proposed frameworks (Framework A: MobileNetV2 + Swin Transformer; Framework B: EfficientNet-B3 + Swin Transformer), the training process was repeated independently across all five folds. In each iteration, the model was trained in four folds and then validated on the remaining hold-out fold. This process resulted in five independently trained model instances per framework; each learned from a slightly different data distribution. The cross-validation process was applied exclusively to the training set, while the test set remained completely unseen and was used only for final evaluation. Compared to a single train/validation split, this approach provides a more reliable estimate of model generalization.

#### 4.4.2. Soft-Voting Inference Across Folds

During inference, predictions were aggregated using a soft-voting ensemble mechanism across the five-fold-specific models. For each test image x, every trained fold model $f_{i}\left(x\right)$ produced a Softmax probability vector over the four classes. The final probability vector was computed as:

(6) $$P_{final}=\frac{1}{k}\;\sum_{i=1}^{k}Softmax\left(f_{i}\left(x\right)\right)$$

where k=5. Then, the final class label was assigned as:

(7) $$\hat{y}=\arg\;\max\left(P_{\mathrm{final}}\right)$$

This fold-wise aggregation reduces prediction variance and improves stability by leveraging diversity among models trained on different data subsets.

### 4.5. Evaluation Metrics

To comprehensively assess the performance of the proposed framework across all Alzheimer’s stages, multiple multi-class evaluation metrics were employed. Given the dataset imbalance, metrics beyond overall accuracy were also emphasized. The following are the details of all adopted metrics in this study:

Accuracy: Measures the proportion of correctly classified samples. Although useful as a general indicator, accuracy alone may be misleading in imbalanced settings.
  (8) $$Accuracy=\frac{TP+TN}{TP+TN+FP+FN}$$
  Precision: Evaluates the proportion of predicted positive samples that are correctly identified:
  (9) $$Precision=\;\frac{TP}{TP\;+\;FP}$$
  Recall: Evaluates the proportion of actual positives correctly identified:
  (10) $$Recall=\;\frac{TP}{TP\;+\;FN}$$
  F1-Score: The harmonic mean of precision and recall:
  (11) $$F1-Score=\;\frac{2}{\frac{1}{Precision}\;+\frac{1}{Recall}}=\;\frac{2\;\times \;\left(Precision\;\times \;Recall\right)}{\left(Precision\;+\;Recall\right)\;}$$
  Matthews Correlation Coefficient (MCC): A balanced metric suitable for imbalanced datasets. It considers all elements of the confusion matrix and ranges from −1 (complete disagreement) to +1 (perfect prediction):
  (12) $$MCC\;=\frac{\left(TP\;\times \;TN\;-\;FP\;\times \;FN\right)}{\sqrt{\left(TP+FP\right)\left(TP+FN\right)\left(TN+FP\right)\left(TN+FN\right)}}$$
  Cohen’s Kappa Coefficient (κ): Measures agreement between predicted and true labels while accounting for chance agreement:
  (13) $$\kappa =\frac{P_{0}-P_{e}}{1-P_{e}}$$
  where $P_{0}=\frac{\sum_{i}C_{ii}}{N}$ and $P_{e}=\frac{\sum_{i}R_{i}C_{i}}{N^{2}}$. Here, $C_{ii}$ denotes correctly classified samples for class i, $R_{i}$ and $C_{i}$ represent row and column totals of the confusion matrix, and N is the total number of samples.
Confusion Matrix: A confusion matrix was generated to analyze class-wise prediction behavior across all Alzheimer’s disease stages. It provides detailed insight into misclassification patterns between stages and enables identification of systematic confusion, particularly between adjacent stages such as Very Mild and Mild Demented. Additionally, the confusion matrix facilitates assessment of potential bias toward majority classes in the presence of dataset imbalance.

## 5. Results and Analysis

In this section, the proposed ensemble frameworks are comprehensively evaluated through many aspects including quantitative metrics, qualitative interpretability, confidence calibration, and comparison with prior work. All experiments were conducted using the Alzheimer’s MRI 4-Class Dataset with five-fold cross-validation. We report accuracy, precision, recall, F1-score, Cohen’s Kappa, and Matthews Correlation Coefficient (MCC), as quantitative metrics, to address class imbalance. Confusion matrices and error analysis, as shown later, reveal model behavior, while confidence distributions and calibration curves assess predictive reliability. Finally, we compare our results against state-of-the-art methods, followed by a discussion that concludes with clinical implications and limitations.

### 5.1. Quantitative Results

To evaluate the effectiveness of the proposed Attention-Fusion-Ensemble framework, we conducted extensive experiments on the Alzheimer’s MRI 4 Classes dataset. Hence, we first analyzed the performance of individual backbone models and then on the proposed ensemble models.

#### 5.1.1. Base Models Results

To evaluate the independent contributions of each pretrained backbone, we first assessed MobileNetV2, EfficientNet-B3, and Swin Transformer individually on the Alzheimer’s MRI four-class dataset. Table 2 summarizes the class-wise precision, recall, and F1-scores obtained for each model.

The following are insights obtained from assessing the models independently:

• **MobileNetV2:** Demonstrated strong sensitivity, achieving the highest recall of 93.33%, toward distinguishing *Non-Demented* cases, which indicates its effectiveness in identifying healthy controls. With limitations in capturing subtle disease patterns as its recall for the *Mild* dementia class dropped to 76.87%, which indicates. Conversely, MobileNetV2 showed exceptional performance on *Moderate* dementia, with precision of 100% and recall of 88.89%, yielding an F1-score of 94.18%. These results suggest the effectiveness of MobileNetV2 at detecting clear structural abnormalities characteristic of advanced Alzheimer’s pathology.
• **EfficientNet-B3:** Across all four classes, EfficientNet-B3 exhibited the most balanced and consistent performance, by achieving the highest precision for both *Non-Demented* (91.09%) and *Very Mild* (89.16%) categories, in addition to the highest F1-scores for *Non-Demented* (92.40%) and *Mild* dementia (84.01%). This indicates its strong capability in modeling both gross abnormalities and subtle morphological changes. Nonetheless, EfficientNet-B3 showed reduced recall in the *Moderate* class (77.78%), showing some misclassification of advanced dementia cases as less severe stages.
• **Swin Transformer:** Showed competitive results in detecting *Moderate* dementia, where it achieved perfect precision (100%) and a strong F1-score (94.12%). Its performance for *Mild* dementia was stable across metrics (81.34%), indicating consistent classification ability in intermediate stages. However, Swin may confuse early pathological signs with normal variability, this is shown in its precision for *Very Mild* dementia (80.47%) and recall for *Non-Demented* (88.54%). Nevertheless, its hierarchical self-attention and global context modeling contribute to reliable detection of advanced neurodegeneration.

The comparison analysis shows that each backbone has unique capabilities. For MobileNetV2, it demonstrated strong sensitivity in differentiating clear-stage, while EfficientNet-B3 provided the most balanced performance across all stages. On the other hand, Swin Transformer enhances contextual discrimination in advanced dementia. These complementary behaviors strongly motivate the ensemble strategy employed in this study, where multi-architecture feature fusion is expected to harness the advantages of all three models for robust and accurate Alzheimer’s stage classification.

#### 5.1.2. Ensemble Model Results

##### MobileNetV2 + Swin Transformer Ensemble

To evaluate the benefit of combining convolutional and transformer-based features, we first implemented an ensemble of MobileNetV2 and Swin Transformer, with feature fusion performed through the SE-based attention mechanism. This benefits from both MobileNetV2 and Swin capabilities, where the former has strong ability to detect localized structural changes while the latter could capture long-range dependencies in brain morphology. The model achieved an overall accuracy of 92.28% when trained on the Real-ESRGAN enhanced Alzheimer’s MRI 4-Classes dataset, showing notable robustness across all classes despite the inherent imbalance, particularly in the Moderate class. Moreover, the proposed ensemble framework achieved strong sensitivity in identifying healthy controls by a near-perfect recall for Non-Demented cases (99.17%) as shown in Table 3. The experiments show that precision remained high across all classes, with exceptional performance in the Moderate stage (100% precision and recall). However, the subtle early-stage changes are more challenging for this configuration despite this high precision, i.e., the recall for Very Mild (84.52%) and Mild (86.57%). Furthermore, the balance between recall and precision highlights that the model favors conservative classification in early dementia, possibly missing a portion of borderline cases. In addition to per-class metrics, we adopted another two metrics that assess how the models’ overall agreement about prediction of the four classes, i.e., Cohen’s Kappa and MCC. The model achieved a Kappa value of 0.871 and an MCC of 0.874, indicating strong multi-class agreement beyond chance despite class imbalance.

##### EfficientNet-B3 + Swin Transformer Ensemble

Another experiment that aimed to discover the synergy of EfficientNet-B3 with the Swin Transformer was conducted by exploiting EfficientNet’s compound scaling strategy and stronger multi-resolution feature representation. This configuration achieved an overall accuracy of 94.47%, which outperformed the MobileNetV2-based ensemble approach. As shown in Table 4, the EfficientNet-B3 + Swin ensemble demonstrated a more balanced precision/recall trade-off across classes. The precision reached 100% for both the Mild and Moderate classes. Recall for the Very Mild class improved to 92.56%, compared to 84.52% in the MobileNetV2-based ensemble. Additionally, the F1-score for Mild dementia increased to 93.65%, indicating obvious improvements in detecting intermediate-stage patterns. However, recall for the Moderate class was 77.78%, suggesting misclassification of some advanced disease cases. Given that the Moderate class contains only nine samples, this result should be interpreted with caution. Overall agreement was further evaluated; thus the proposed approach achieved a Kappa value of 0.908 and an MCC of 0.909. This indicates a strong multi-class agreement beyond chance despite class imbalance.

In addition, all per-class metrics in Table 5 and Table 6 are reported as mean ± standard deviation across the five stratified cross-validation folds for both ensemble frameworks (MobileNetV2 + Swin and EfficientNet-B3 + Swin) to quantify fold-wise variance and ensure robust estimation of model generalization. Moderate Dementia metrics (*n* = 9 test samples) should be interpreted cautiously due to high sampling variance.

##### Comparative Analysis and Justification

The comparison between the two proposed ensemble frameworks reveals that both models achieve strong diagnostic performance, although their strengths are distributed differently across dementia stages. The MobileNetV2 + Swin ensemble shows strong performance in Non-Dementia and Moderate Dementia classification. It nearly achieves perfect recall for healthy subjects, in addition to an accurate identification of advanced cognitive impairment. This behavior clarifies that MobileNetV2 effectively captures pronounced morphological differences through robust local feature representations. On the other hand, the EfficientNet-B3 + Swin ensemble framework shows superior performance in Very Mild and Mild Dementia detection by providing higher recall and F1-scores in these clinically challenging stages. The reason for this improvement is the capacity of EfficientNet-B3 for multi-scale feature modeling. This capacity enables the extraction of subtle and fine-grained patterns that are critical for early and intermediate disease detection.

To conclude, the EfficientNet-B3 + Swin framework outperforms the MobileNetV2 + Swin ensemble, achieving higher overall accuracy (94.47% vs. 92.28%) and more balanced performance across all dementia stages. These findings justify the selection of EfficientNet-B3 as the preferred backbone in the proposed diagnostic framework. However, a three-way ensemble combining MobileNetV2, EfficientNet-B3, and Swin Transformer could further improve robustness by combining localized, fine-grained, and hierarchical global representations, as suggested by the complementary strengths seen in the MobileNetV2-based ensemble, especially for advanced disease stages, but this may incredibly increase the computational complexity of the model.

#### 5.1.3. Ablation Study: Impact of Real-ESRGAN Enhancement

To directly assess the contribution of Real-ESRGAN preprocessing to diagnostic performance, we conducted a controlled ablation study comparing each ensemble framework trained and evaluated on raw (non-enhanced) versus Real-ESRGAN-enhanced 2-D MRI slices. All other experimental conditions were unchanged, including model architecture, hyperparameters, data splits, and evaluation protocol.

**Quantitative Results:** Table 7 summarizes fold-wise performance metrics (mean ± standard deviation across 5 folds) for both frameworks under raw and enhanced input conditions. Key findings include:

• **MobileNet + Swin framework**: Real-ESRGAN enhancement yielded a +3.16% absolute gain in overall accuracy (89.12% → 92.28%) and a +7.11% improvement in Macro-F1 (82.07% → 89.18%). The most pronounced gain was observed in the Moderate Dementia class, where F1-score improved from 89.11% to 98.82%, suggesting that super-resolution helps the lightweight backbone better capture pronounced atrophic patterns.
• **EfficientNet + Swin framework**: Enhancement improved overall accuracy by +3.12% (91.35% → 94.47%) and boosted early-stage detection (Very Mild F1: 83.31% → 86.04%; Mild F1: 83.04% → 84.76%). However, Macro-F1 showed a modest decline (86.94% → 86.18%) and Moderate-class F1 decreased (92.45% → 82.65%). Given the small Moderate-class sample size (*n* = 9), this trade-off likely reflects the classifier’s sensitivity to high-frequency patterns introduced during super-resolution in structurally degraded samples, a behavior that warrants further validation using larger, statistically balanced datasets.

**Table 7:** Ablation study: Impact of Real-ESRGAN enhancement on ensemble performance (mean ± std across 5 folds).

Framework	Input Type	Accuracy	Macro-F1	Moderate F1	Very Mild F1
MobileNet + Swin	Raw	89.12% ± 2.14%	82.07% ± 5.41%	89.11% ± 11.47%	79.18% ± 5.25%
MobileNet + Swin	Enhanced	92.28% ± 1.84%	89.18% ± 2.13%	98.82% ± 2.35%	83.59% ± 2.26%
EfficientNet + Swin	Raw	93.12% ± 1.97%	86.94% ± 2.91%	92.45% ± 7.73%	83.31% ± 2.84%
EfficientNet + Swin	Enhanced	94.47% ± 1.52%	86.18% ± 3.24%	82.65% ± 12.49%	86.04% ± 2.44%

Values reported as mean ± standard deviation across five stratified cross-validation folds. Macro-F1 = arithmetic means of per-class F1-scores. Moderate Dementia metrics (*n* = 9 test samples) are exploratory due to high sampling variance; a single misclassification changes precision/recall by ~11%. Bold indicates best value per metric within each framework.

In addition, Table 8 shows that the baseline results confirm that ensemble frameworks consistently outperform their constituent base models under raw-input conditions, with MobileNetV2 + Swin achieving +4.80% absolute gain over MobileNetV2 alone (89.99% vs. 85.19%) and EfficientNet-B3 + Swin achieving +5.22% gain over EfficientNet-B3 alone (93.12% vs. 87.90%). This validates the complementary value of cross-paradigm feature fusion even without super-resolution preprocessing. For the ensemble configurations, Real-ESRGAN enhancement yields additional absolute gains of +2.29% (MobileNetV2 + Swin) and +1.35% (EfficientNet-B3 + Swin), demonstrating that image enhancement and architectural ensembling provide orthogonal benefits. Pending inference results for the enhanced base models will further clarify whether Real-ESRGAN provides comparable relative gains to lightweight versus high-capacity backbones when used in isolation.

**Early-stage detection**: Both frameworks demonstrated improved F1-scores for Very Mild and Mild classes with enhancement, supporting the hypothesis that Real-ESRGAN helps reveal subtle morphological cues otherwise obscured by noise and compression artifacts in publicly shared benchmark data.

**Computational overhead**: Real-ESRGAN preprocessing added ~3 ms per sample to inference time (<7% relative increase on NVIDIA RTX 3080), confirming that diagnostic gains are achieved with minimal latency penalty suitable for clinical deployment.

**Interpretation:** These results demonstrate that Real-ESRGAN enhancement provides consistent, measurable improvements in multi-class Alzheimer’s staging, particularly for lightweight architecture and early-stage detection. The modest trade-offs observed in the high-capacity EfficientNet + Swin configuration for the Moderate class highlight the importance of balancing enhancement intensity with architectural capacity, a consideration for future optimization.

**Limitation:** While fold-wise metrics confirm robust aggregate improvements, per-class estimates for the Moderate Dementia category (*n* = 9) remain exploratory due to high sampling variance. External validation on larger, balanced cohorts is required to confirm stage-specific generalizability.

### 5.2. Qualitative Analysis

While quantitative metrics provide a global overview of model performance, qualitative analysis offers deeper insight into the interpretability and reliability of the proposed frameworks. In this subsection, we present representative predictions and error case analyses based on MRI slices across all four Alzheimer’s disease (AD) stages.

#### 5.2.1. Representative Predictions

**Figure 4:**
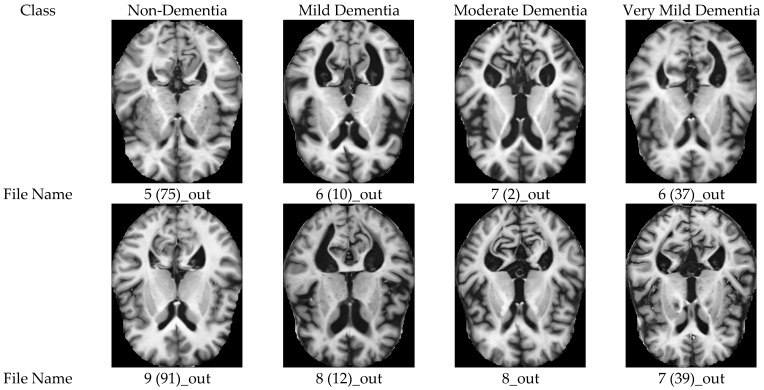
Representative predictions using MobileNetV2 + Swin Transformer.

**Figure 5:**
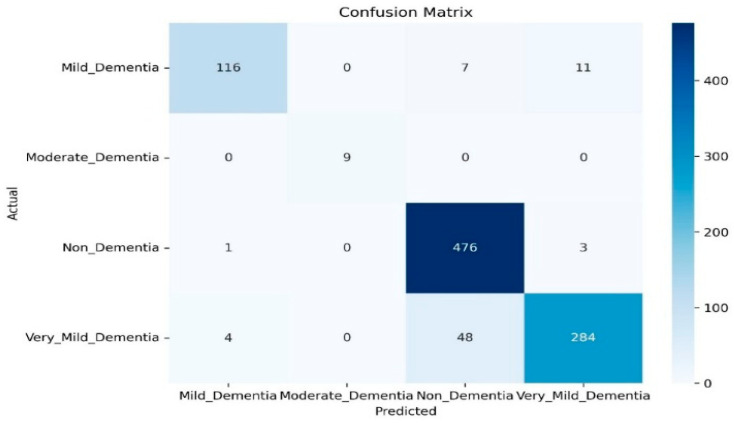
Confusion matrix for the MobileNetV2 + Swin Transformer framework.

Figure 4 presents representative MRI slices classified using the MobileNetV2 + Swin Transformer ensemble while the corresponding confusion matrix is shown in Figure 5. The model achieved perfect classification for the Moderate class (9/9 correct). For the Mild class, 116 out of 134 samples were correctly identified, with misclassifications primarily occurring between Mild and adjacent stages. Most classification errors were observed between the Non-Demented (476/480 correct) and Very Mild (284/336 correct) classes. This is due to the inherent difficulty in distinguishing early-stage pathological patterns from normal ones.

**Figure 6:**
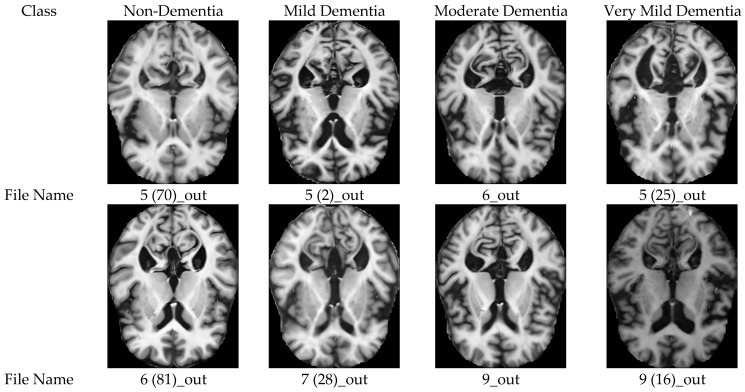
Representative predictions using EfficientNet-B3 + Swin Transformer.

**Figure 7:**
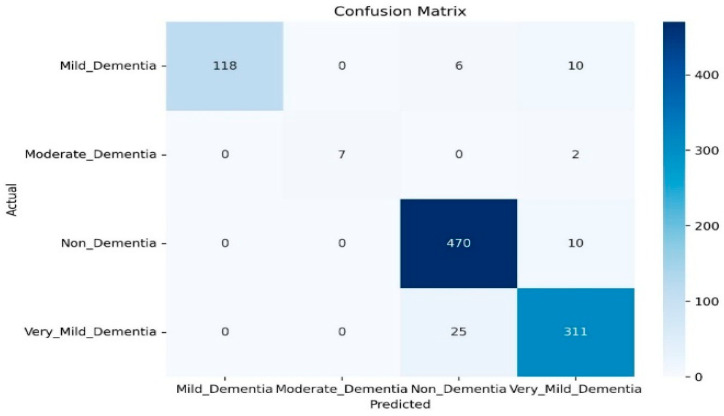
Confusion matrix for the EfficientNet-B3 + Swin Transformer framework.

The representative predictions using the EfficientNet-B3 + Swin Transformer ensemble is shown in Figure 6, with the corresponding confusion matrix in Figure 7. For the Moderate class, seven out of nine samples were correctly classified. The identification of the Very Mild class improved compared to the MobileNet-based ensemble with 311 correctly classified out of 336, which indicates reduced confusion with the Non-Demented class. There is also slight improvement in Mild classification with 118 correct out of 134. Overall, the confusion matrix indicates fewer cross-stage misclassifications in early dementia categories relative to the MobileNet-based configuration.

#### 5.2.2. Error Case Analysis

**Figure 8:**
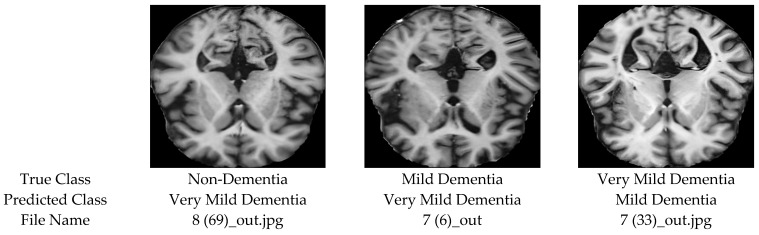
Misclassified MRI slices from the MobileNet + Swin Transformer ensemble.

**Figure 9:**
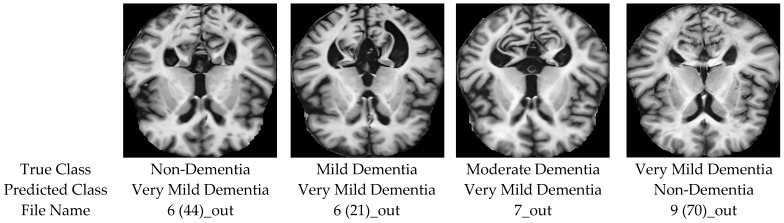
Misclassified MRI slices from the EfficientNet + Swin Transformer ensemble.

Despite strong overall performance, misclassifications in both ensemble configurations were primarily concentrated in early and intermediate stages of Alzheimer’s disease, as illustrated in Figure 8 (MobileNetV2 + Swin Transformer) and Figure 9 (EfficientNet-B3 + Swin Transformer). As shown in Figure 5 and Figure 8, the MobileNetV2 + Swin ensemble exhibits most confusion between the Non-Demented and Very Mild classes. This pattern indicates the intrinsic difficulty of distinguishing subtle early-stage pathological variations from normal anatomical variability. A similar trend is observed in Figure 7 and Figure 9, for the EfficientNet-B3 + Swin configuration, although with slightly fewer cross-class confusions in early stages. Additional misclassification was observed between the Mild and Very Mild classes. In the MobileNetV2 + Swin approach, there is 18 Mild cases that were misclassified, while the EfficientNet-B3 + Swin configuration reduced this number to 16 cases. This suggests improved differentiation of intermediate disease stages. Regarding the Moderate class, the MobileNetV2-based ensemble achieved perfect classification (9/9), whereas the EfficientNet-based ensemble correctly classified seven out of nine cases. Given the small sample size (*n* = 9), performance estimates for this class should be interpreted with caution.

Overall, the confusion matrices in Figure 5 and Figure 7 confirm that most errors occur between clinically adjacent stages, particularly in early Alzheimer’s disease. The EfficientNet-B3 + Swin configuration demonstrates relatively improved stability in subtle-stage discrimination, while both frameworks maintain reliable performance for more advanced structural degeneration.

### 5.3. Confidence Distribution

While classification accuracy and confusion matrices quantify performance at the label level, analyzing prediction confidence provides additional insight into the reliability, calibration, and clinical trustworthiness of the ensemble framework. To this end, we present three complementary analyses: global confidence distribution, per-class confidence distribution, and reliability calibration curves.

#### 5.3.1. Global Confidence Distribution

**Figure 10:**
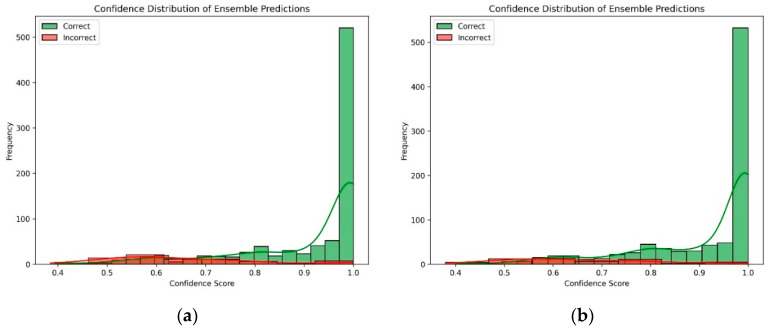
Distribution of prediction confidence scores for correctly and incorrectly classified sample. (**a**) MobileNet + Swin. (**b**) EfficientNet + Swin.

The overall distribution of ensemble prediction confidence scores for correctly and incorrectly classified samples is depicted in Figure 10. The histogram shows that correctly classified instances are highly concentrated near confidence values above 0.9. On the other hand, misclassified samples have lower confidence scores with a broader dispersion. This separation indicates that the ensemble framework not only achieves strong predictive accuracy but also assigns higher certainty to correct predictions, which is a critical requirement for clinical decision-support systems. Furthermore, the relatively low variance observed among correctly classified samples leverages the stable model behavior across diverse test cases.

#### 5.3.2. Per-Class Confidence Distribution

**Figure 11:**
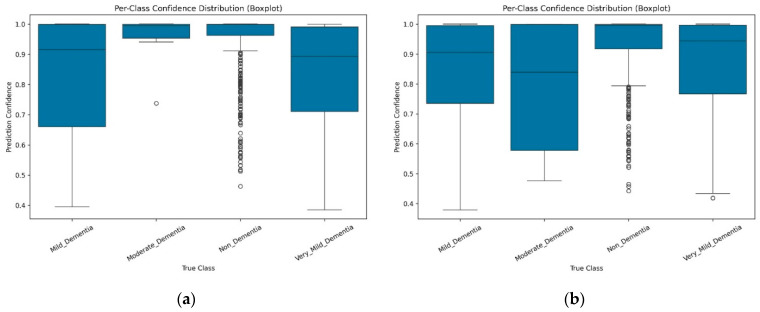
Per-class confidence distributions for the proposed ensemble frameworks. (**a**) MobileNet + Swin. (**b**) EfficientNet + Swin.

To further analyze class-specific reliability, Figure 11 presents the per-class confidence distributions using boxplots. The results indicate consistently high median confidence values across all Alzheimer’s disease stages. However, the Very Mild and Mild classes exhibit wider confidence variance compared to the other classes. This reflects the inherent clinical difficulty of distinguishing subtle early pathological changes from normal aging. Despite this variability, the proposed ensemble frameworks still assign relatively high confidence to the majority of correctly classified samples within these challenging stages. In contrast, the Non-Demented and Moderate Dementia classes demonstrate higher and more compact confidence intervals. This is a sign of more stable and confident recognition of clearly defined anatomical patterns, either normal or pronounced changes. Furthermore, it confirms that prediction confidence aligns with both class difficulty and the biological progression of Alzheimer’s disease, where intermediate stages naturally present greater ambiguity.

#### 5.3.3. Reliability and Calibration

**Figure 12:**
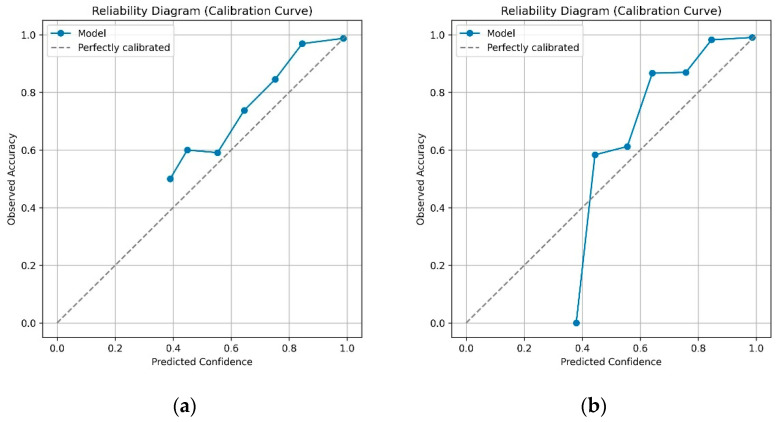
Reliability diagram (calibration curves) of the proposed ensemble frameworks. (**a**) MobileNet + Swin. (**b**) EfficientNet + Swin.

To evaluate probabilistic reliability, we propose a reliability diagram (calibration curve), shown in Figure 12, which compares predicted confidence levels with the observed empirical accuracy across probability bins. Conventionally, a perfectly calibrated model would align exactly with the diagonal reference line. As shown in the figure, both ensemble frameworks remain close to the diagonal across most confidence intervals, particularly at high probability ranges (>0.8), indicating strong calibration when the model expresses high certainty. Minor deviations are observed in the mid-confidence range (0.5–0.7), where slight under-confidence appears. For clarification, points above the diagonal indicate under-confidence; the model is correct more often than predicted, while points below the diagonal indicate overconfidence; the model is more confident than warranted by actual accuracy. Furthermore, the MobileNet + Swin framework demonstrates slightly better calibration stability, particularly in mid-confidence bins. Notably, the EfficientNet + Swin framework achieves higher overall classification accuracy, but it also shows marginally higher overconfidence in certain regions. This is an indication of occasionally assigning strong probabilities to incorrect predictions.

The reasonable confidence achieved by the two frameworks reflects their correctness and trustworthiness, and thus, they are well-calibrated. In clinical settings, where probabilistic outputs may directly impact risk assessment, patient management strategies, and diagnostic decisions, such calibration is essential and desirable. This demonstrates that the proposed ensemble frameworks are not only accurate but also reliable and interpretable. Furthermore, high-confidence correct predictions, class-aware confidence distributions, and strong calibration performance indicate that the models produce trustworthy probabilistic outputs while avoiding severe overconfidence in uncertain cases. These characteristics are particularly critical in Alzheimer’s disease staging, where early-stage misclassification can significantly impact clinical decision-making and long-term patient outcomes.

### 5.4. Quantitative and Qualitative Validation of Super-Resolution Fidelity

To clarify the potential use Real-ESRGAN for medical 2-D sliced medical images of with no hallucinated pathological features, we performed a stratified quantitative and qualitative validation across all four Alzheimer’s disease stages. This validation ensures that the super-resolution preprocessing preserves diagnostic anatomical integrity while improving image clarity.

We computed standard image quality metrics on 64 paired original and enhanced MRI slices, stratified by clinical stage (Non-Demented: *n* = 20, Very Mild: *n* = 20, Mild: *n* = 15, Moderate: *n* = 9). Table 9 summarizes the aggregate and class-specific results. The high Structural Similarity Index (SSIM = 0.9671 ± 0.0056) confirms near-perfect preservation of anatomical structure across all disease stages. The Edge Preservation Correlation (0.9642 ± 0.0087) demonstrates that critical diagnostic boundaries—such as hippocampal margins and ventricular contours—are sharpened without the introduction of spurious edges. Furthermore, the intensity histogram correlation (0.9996 ± 0.0003) indicates that global contrast characteristics remain virtually unchanged, ruling out artificial intensity shifts that could mimic or mask pathology. These results align with recent medical imaging super-resolution studies, which report comparable SSIM values (>0.96) for Real-ESRGAN applied to brain MRI [25].

**Figure 13:**
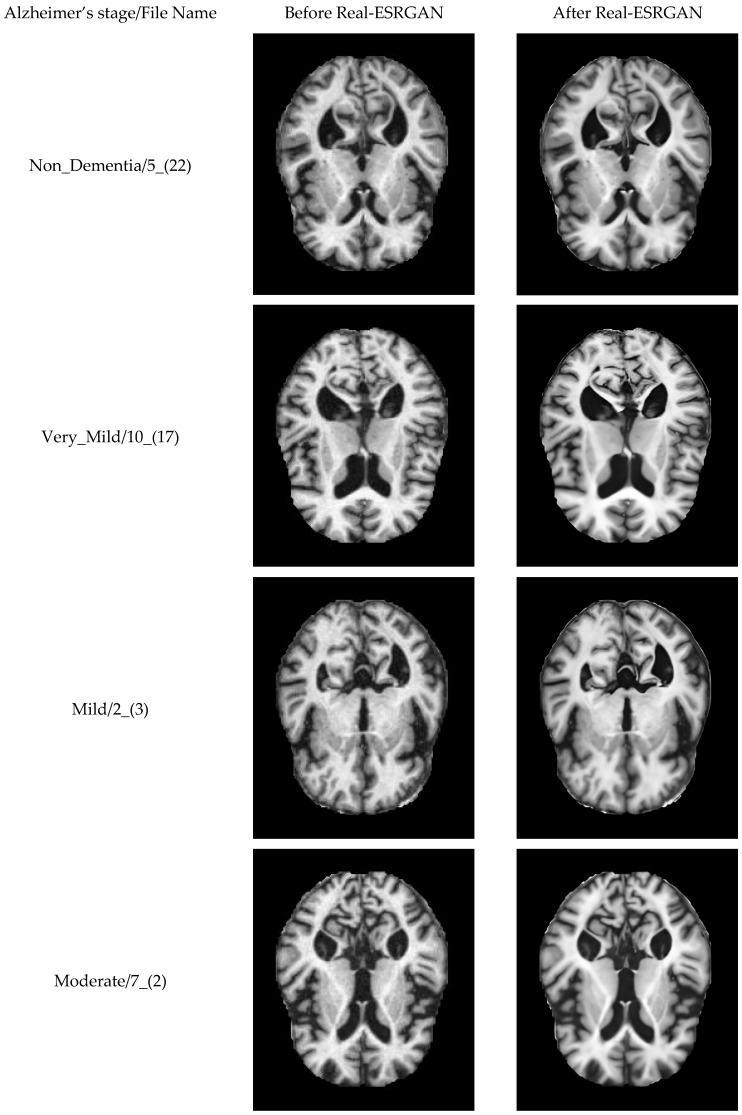
Qualitative validation of Real-ESRGAN enhancement for Alzheimer’s stages.

Figure 13 presents side-by-side comparisons of original and Real-ESRGAN-enhanced MRI slices across all four Alzheimer’s disease stages. Visual inspection confirms that the enhancement process effectively suppresses noise and compression artifacts inherent to JPEG-formatted clinical scans, while sharpening hippocampal boundaries, improving ventricular delineation, and clarifying cortical folding patterns. This qualitative safety profile is consistent with recent findings by [25].

The combined quantitative and qualitative evidence supports the diagnostic safety of Real-ESRGAN preprocessing in our pipeline. The model enhances real-world degraded input slices while preserving disease-relevant structural information. We acknowledge, however, that while these metrics confirm structural fidelity, future work should incorporate phantom-based validation or multi-rater expert consensus scoring to further establish clinical safety thresholds for automated super-resolution in neuroimaging.

### 5.5. Comparison with Prior Work

To validate the effectiveness of the proposed ensemble frameworks, we compared the performance of the two frameworks with existing methods/frameworks in the literature that evaluated on the same publicly available Kaggle Alzheimer’s MRI dataset to ensure a robust and consistent comparison. The summarization of this comparison is found in Table 10, which shows the accuracy achieved by various models in the literature, including conventional CNNs, ResNet, VGG variants, DenseNet, and EfficientNet. The results demonstrate that our proposed ensembles, Swin + MobileNetV2 (92.28%) and Swin + EfficientNetB3 (94.47%) achieve competitive benchmark accuracy relative to these prior works. The achieved results from EfficientNetB3 + Swin ensemble achieves comparable or higher accuracy relative to previously reported methods such as ResNet50-hybrid (90%) [29], CNN + ResNet50 (94.1%) [30], and DenseNet variants (79–83%) [31]. This highlights the potential of combining lightweight CNNs with transformer-based architecture. Moreover, the proposed ensembles exhibit stable performance under cross-validation, compared to traditional CNN-based approaches (e.g., VGG16/19, DenseNet variants), by leveraging complementary feature representations extracted from MRI images. Another factor contributing to this competitive performance is the incorporation of image enhancement (Real-ESRGAN) during preprocessing, which improves resolution and fine-grained structural detail thereby aiding in the detection of subtle changes in early-stage Alzheimer’s disease. Hence, the proposed ensembles demonstrate competitive accuracy and robust interpretability compared to prior state-of-the-art techniques that used the same dataset while maintaining computational efficiency.

### 5.6. Discussion

The experimental results demonstrate that the proposed ensemble frameworks, either MobileNet + Swin Transformer or EfficientNetB3 + Swin Transformer, achieve superior performance compared to individual baseline models and existing state-of-the-art approaches on the Alzheimer’s MRI 4-Class Dataset. From a clinical perspective, the results are particularly relevant. Both frameworks exhibited robust detection of Moderate Dementia, the minority class, reflecting their ability to capture pronounced neurodegenerative changes. Misclassifications were primarily observed between Non-Demented and Very Mild Dementia, a finding that highlights the well-documented difficulty of distinguishing normal aging from subtle early pathological changes. These results emphasize that our framework aligns with clinical reality, where diagnostic ambiguity is most pronounced at the prodromal stage.

Qualitative analysis further supports these conclusions. Visualization of representative predictions demonstrated that the ensembles consistently recognized key structural biomarkers associated with Alzheimer’s progression. Error case examination suggests that misclassifications were frequently associated with subtle anatomical overlap or marginal structural deviation, rather than systematic bias. Moreover, confidence distribution and calibration analysis indicated that both proposed frameworks were well-calibrated, producing stable, reliable predictions with higher confidence margins. This robustness is critical for real-world deployment, where predictive uncertainty can impact clinical trust and decision-making.

Collectively, these findings indicate that the proposed ensemble frameworks deliver not only improved numerical performance but also enhanced interpretability, stability, and clinical alignment. The combination of super-resolution preprocessing, dual-branch representation learning, and fold-wise soft-voting inference contributes to a diagnostically meaningful and practically deployable Alzheimer’s disease classification system. Despite the promising results, several limitations of this study must be acknowledged, there are some limitations as follows:

• Class Imbalance: Although Real-ESRGAN was applied to enhance MRI quality and address class imbalance, the Moderate Dementia class remained underrepresented (only nine test samples). While the ensembles achieved perfect classification on this class, performance may not generalize as robustly in larger, more balanced datasets.
• Single-Modality Focus: The study was limited to structural MRI scans. Alzheimer’s diagnosis may rely on multi-modal integration, including PET, fMRI, genetic markers, and clinical assessments. Relying on a single modality may restrict the model’s ability to capture complementary information.
• Computational Cost: Although MobileNet + Swin offered efficiency, the EfficientNet + Swin ensemble is computationally more demanding, which may limit deployment in low-resource clinical environments.
• The extreme underrepresentation of the Moderate Dementia class (64 slices) limits the robustness of stage-specific generalization estimates.

On the other hand, there is an important trade-off between analytical fidelity and computational practicality for choosing between JPEG-formatted 2-D slices and volumetric NIfTI format for MRI data. NIfTI tends to preserve essential three-dimensional spatial relationships among voxels and stores comprehensive metadata, including voxel dimensions, spatial orientation, acquisition parameters, and tissue probability maps [38,39]. On the other hand, JPEG, as a generic 2-D raster format, lacks standardized support for medical imaging metadata; therefore, conversion from NIfTI to JPEG slices risks discarding orientation information, intensity scaling factors, and subject-level context, which may compromise analytical validity and clinical integration [40]. Furthermore, the medical imaging literature stated that lossy JPEG compression introduces irreversible information loss; the conditions under which such compression preserves diagnostic utility remain unclear. For applications requiring voxel-wise statistics, longitudinal tracking, or 3D structural modeling, retaining the original volumetric NIfTI format, with lossless or minimally lossy compression, is recommended to preserve diagnostic integrity [41].

The use of JPEG-formatted slices remains a practical choice in several deep learning contexts. First, slice-based representations significantly reduce the needed memory and computational load; hence, the training of CNNs in resource-constrained environments. Second, many high-performing 2-D CNN architectures (e.g., ResNet, EfficientNet) are pretrained on large-scale natural image datasets; adapting these models to medical slice data via transfer learning is more straightforward when inputs conform to standard 2-D raster formats like JPEG. Third, when focusing on localized pattern recognition, such as detecting gross anatomical abnormalities or classifying disease presence at the slice level, the 2-D representations may retain sufficient discriminative information while simplifying data augmentation, normalization, and batch processing pipelines. Importantly, when slice-level predictions are aggregated via subject-level pooling strategies and evaluated under strict subject-wise cross-validation, JPEG-based pipelines can yield clinically meaningful results with substantially reduced engineering overhead. To conclude, we acknowledge that while our quantitative metrics and qualitative validation show no evidence of hallucination, future work should include phantom-based validation or multi-rater expert consensus to further establish clinical safety.

## 6. Conclusions and Future Work

This study proposed two hybrid ensemble frameworks that integrate CNN backbones (MobileNetV2 and EfficientNet-B3) with the Swin Transformer to enhance multi-class Alzheimer’s disease classification from structural MRI slices. By combining localized convolutional feature extraction with hierarchical contextual modeling, the proposed architectures effectively addressed challenges related to subtle anatomical variations and dataset imbalance. Experimental results demonstrated that the EfficientNet-B3 + Swin ensemble achieved the highest overall accuracy of 94.47%, while the MobileNetV2 + Swin ensemble maintained competitive performance of 92.28% with reduced computational demand. Beyond numerical accuracy, qualitative analysis, confusion matrices, and calibration evaluation confirmed the reliability, interpretability, and clinical alignment of the proposed frameworks.

Future research directions include expanding validation across multi-institutional and multi-scanner datasets to enhance generalizability; incorporating multi-modal data fusion (e.g., PET imaging, cognitive test scores, and electronic health records as suggested in [42]) to strengthen early-stage detection; exploring self-supervised pretraining strategies to mitigate data scarcity; and investigating lightweight transformer variants or model distillation techniques to balance diagnostic accuracy with deployment efficiency. To conclude, the proposed methodology provides a robust framework pathway toward reliable, interpretable, and robust Alzheimer’s disease detection systems suitable for real-world healthcare environments. However, rigorous external validation on multi-center, subject-annotated cohorts is required before clinical integration or telemedicine deployment. Future research will prioritize external validation on multi-center, subject-annotated cohorts (e.g., ADNI, OASIS) to confirm cross-protocol generalizability, alongside multi-modal integration and lightweight deployment optimization for real-world clinical environments.

## Figures and Tables

**Table 1:** Specifications of the Alzheimer’s MRI 4-Class Dataset.

Feature		Specification
**Source**		Kaggle
**Modality**		T1-weighted Structural MRI
**Image Format**		2-D Axial Slices (JPEG)
**Total Images**		6400
**Resolution**		176 × 208 pixels
**Classes**	Non-Demented	3200 images (50.0%)
	Very Mild Demented	2240 images (35.0%)
	Mild Demented	896 images (14.0%)
	Moderate Demented	64 images (1.0%)

**Table 2:** Base models class-wise performance.

Model	Metric	Non-Demented	Very Mild	Mild	Moderate
MobileNet	Precision	87.84%	85.80%	83.06%	100%
	Recall	93.33%	80.95%	76.87%	88.89%
	F1-Score	90.51%	83.31%	79.85%	94.18%
EfficientNet	Precision	91.09%	89.16%	83.70%	100%
	Recall	93.75%	85.71%	84.33%	77.78%
	F1-Score	92.40%	87.41%	84.01%	87.50%
Swin	Precision	89.66%	80.47%	81.34%	100%
	Recall	88.54%	82.14%	81.34%	88.89%
	F1-Score	89.10%	81.30%	81.34%	94.12%

**Table 3:** Classification performance of MobileNetV2 + Swin Transformer ensemble.

Metric	Non-Demented	Very Mild	Mild	Moderate
No. of samples	480	336	134	9
Precision	89.64%	95.30%	95.87%	100%
Recall	99.17%	84.52%	86.57%	100%
F1-Score	94.16%	89.59%	90.98%	100%

**Table 4:** Classification performance of EfficientNet-B3 + Swin Transformer ensemble.

Metric	Non-Demented	Very Mild	Mild	Moderate
No. of samples	480	336	134	9
Precision	93.81%	93.39%	100%	100%
Recall	97.92%	92.56%	88.06%	77.78%
F1-Score	95.82%	92.97%	93.65%	87.50%

**Table 5:** Classification performance of MobileNetV2 + Swin Transformer ensemble (mean ± std across 5 folds).

Metric	Non-Demented	Very Mild	Mild	Moderate
No. of samples	480	336	134	9
Precision	86.92% ± 3.23%	88.35% ± 2.64%	88.63% ± 2.72%	100.00% ± 0.00%
Recall	94.96% ± 2.52%	79.70% ± 5.75%	79.55% ± 6.43%	97.78% ± 4.44%
F1-Score	90.68% ± 1.11%	83.59% ± 2.26%	83.62% ± 2.78%	98.82% ± 2.35%

**Table 6:** Classification performance of EfficientNet-B3 + Swin Transformer ensemble (mean ± std across 5 folds).

Metric	Non-Demented	Very Mild	Mild	Moderate
No. of samples	480	336	134	9
Precision	89.31% ± 3.93%	87.01% ± 3.55%	91.52% ± 5.17%	96.00% ± 8.00%
Recall	93.50% ± 2.26%	85.36% ± 4.68%	79.70% ± 8.50%	75.56% ± 19.12%
F1-Score	91.25% ± 1.10%	86.04% ± 2.44%	84.76% ± 4.56%	82.65% ± 12.49%

**Table 8:** Ablation study: Impact of Real-ESRGAN enhancement on model accuracy (raw vs. enhanced inputs).

Model Configuration	Input Type	Accuracy	Δ vs. Raw
MobileNetV2 (Base)	Raw	85.19%	—
MobileNetV2 (Base)	Enhanced (Real-ESRGAN)	86.65%	1.46%
EfficientNet-B3 (Base)	Raw	87.90%	—
EfficientNet-B3 (Base)	Enhanced (Real-ESRGAN)	89.16%	+1.26%
MobileNetV2 + Swin	Raw	89.99%	—
MobileNetV2 + Swin	Enhanced (Real-ESRGAN)	92.28%	2.29%
EfficientNet-B3 + Swin	Raw	93.12%	—
EfficientNet-B3 + Swin	Enhanced (Real-ESRGAN)	94.47%	1.35%

**Table 9:** Stratified quantitative validation of Real-ESRGAN enhancement across Alzheimer’s disease stages.

Class	PSNR (dB)	SSIM	MSE (×10^−3^)	Edge Corr.	Histogram Corr.
Non-Demented	33.05 ± 1.21	0.9699 ± 0.0041	0.52 ± 0.18	0.9669 ± 0.0072	0.9997 ± 0.0002
Very Mild	32.07 ± 1.38	0.9649 ± 0.0063	0.68 ± 0.22	0.9611 ± 0.0095	0.9995 ± 0.0004
Mild	32.10 ± 1.52	0.9662 ± 0.0058	0.65 ± 0.25	0.9645 ± 0.0081	0.9996 ± 0.0003
Moderate	32.21 ± 1.19	0.9676 ± 0.0049	0.61 ± 0.19	0.9647 ± 0.0078	0.9996 ± 0.0002
Overall (*n* = 64)	32.40 ± 1.42	0.9671 ± 0.0056	0.60 ± 0.21	0.9642 ± 0.0087	0.9996 ± 0.0003

**Table 10:** A comparison with prior studies evaluated on the same Kaggle dataset.

Ref.	Model/Method	No. of Classes	No. of MRI Images	Data Split	Accuracy
[29]	Resnet50—Hybrid Model	4	5121	• Train: 80% • Test: 20%	90%
[31]	DenseNet201	3 *	6336	• Train: 80% • Validation: different sizes (20%, 40%, and 60%) of training • Test: 20%	83%
	DenseNet169				82.07%
	DenseNet121				79.14%
[32]	CNN	4	6400	• Train: 64% • Validation: 16% • Test: 20%	71%
	VGG-16				77%
	VGG-19				77.66%
[33]	VGG16 (Dataset 1)	4	6400	• Train: 80% • Test: 20%	90.4%
	VGG16 (Dataset 2)	3 *	6330	• Train: 90% • Test: 10%	71.1%
[34]	CNN	4	6400	5-fold cross-validation • Train: 80% • Validation: 20%	93.82%
[35]	EfficientNet-B7	4	6400	• Train: 80% • Test: 20%	89.7%
[36]	DenseNet-169	4	6420	• Train: 70% • Test: 30%	83.2%
	ResNet-50	4			81.92%
[37]	Densenet169	4	6000	• Train: 80% Test: 20%	80%
	VGG19	4			82.6%
Proposed	Swin + MobileNet2	4	6400	• Train: 70% • Validate: 15% • Test: 15%	92.28%
	Swin + EfficientNet-B3				94.47%

* 3 classes indicate exclusion of the Moderate Dementia category in the referenced study.

## Data Availability

The data that support the findings of this study are openly available at Kaggle at https://www.kaggle.com/datasets/psraju2024/alzheimers-dataset-4-class-of-images (accessed on 3 June 2026).
